# The effect of body position on cardiovascular, skeletal muscle and ventilatory responses to submaximal cycling

**DOI:** 10.1113/EP092256

**Published:** 2024-12-17

**Authors:** Robert F. Bentley, Jonaline B. Bernal, Daniel C. Basile, Adam N. Di Salvo, Jacob L. Schwartz

**Affiliations:** ^1^ Faculty of Kinesiology & Physical Education University of Toronto Toronto Ontario Canada

**Keywords:** cardiac output, exercise, oxygen consumption, perceived exertion, semi‐upright, skeletal muscle saturation

## Abstract

The completion of exercise in different body positions can impact the function of various components of the oxygen delivery pathway; however, the effect of the haemodynamic conditions induced by a semi‐upright body position on the integrative physiological response to exercise is poorly understood. The purpose of this study was to explore the effect of a semi‐upright body position on cardiac output (CO), vastus lateralis oxygen saturation ($S_{mO_{2}}$), oxygen consumption (${\dot{V}}_{O_{2}}$) and ratings of perceived exertion (Borg RPE) during submaximal cycling. Twenty healthy individuals (22 ± 3 years, 50% female) each completed alternating 5‐min bouts of submaximal upright and semi‐upright (40° incline) cycling at 50 and 100 W. CO, $S_{mO_{2}}$, ${\dot{V}}_{O_{2}}$ and RPE were assessed at rest and at each exercise intensity during steady state. There was a main effect of intensity on the increase in CO, $S_{mO_{2}}$, ${\dot{V}}_{O_{2}}$ and RPE (all *P *< 0.001). In a semi‐upright position, the increase in CO (7.9 ± 2.8 vs. 6.4 ± 2.6 L/min, *P *< 0.001), RPE (median (interquartile range): 11 (9–13) vs. 10 (8–12), *P* = 0.013) and the decrease in $S_{mO_{2}}$ (−38 ± 23 vs. −21% ± 18%, *P *< 0.001) were greater than upright, while the increase in ${\dot{V}}_{O_{2}}$ was attenuated (1.030 ± 0.130 vs. 1.154 ± 0.165 L/min, *P *< 0.001). These results suggest that while a semi‐upright body position produces elevations in CO, these elevations do not seem to perfuse the active skeletal muscle. This may explain the elevation in RPE despite a blunting in the increase in ${\dot{V}}_{O_{2}}$. Further work is required to understand the effects of a semi‐upright exercise position on skeletal muscle activation and lower limb blood flow.

## Abstract (highlights)

- **What is the central question of this study?**
  What is the effect of a semi‐upright body position on the cardiovascular, skeletal muscle and ventilatory responses to submaximal cycling?
- **What is the main finding and its importance?**
  While semi‐upright cycling produces a greater elevation in cardiac output, this elevation does not seem to perfuse active skeletal muscle as desaturation increases more compared to upright cycling. Further, despite an attenuated increase in the rate of oxygen consumption in the semi‐upright position, perceived exertion and dyspnoea are elevated. These considerations are important when interpreting physiological responses in a semi‐upright exercise position.

## 1 INTRODUCTION

The completion of physical activity is required for activities of daily living to be performed and plays an important role in our quality of life (Edemekong et al., 2022). In order to complete these activities, the cardiovascular system delivers oxygen to our working muscles for internal respiration while external respiration at the lung saturates our blood with oxygen. Detailed and rigorous assessments of cardiac function and the contribution of pulmonary vasculature to disease during aerobic exercise, a relevant physiological stressor, typically employ cycling in a semi‐upright position in order to facilitate stress echocardiography (Sicari et al., 2008) and exercise right heart catheterization (Bentley et al., 2020). However, there is a paucity of data describing the integrative physiological response to semi‐upright cycling, and thus an appropriate and relevant interpretation in such circumstances is incomplete at best. Semi‐upright contrasts with a typical upright cycling position and this difference in body position is of particular interest given the disparate central and peripheral haemodynamic conditions induced by gravity (Hinghofer‐Szalkay, 2011). While exercise in an upright position impairs venous return to the heart, the local pressure gradient for active muscle leg perfusion is enhanced compared to a supine position in which venous return may be improved, but active muscle leg perfusion pressure may be compromised (Gauer, 1965; Sundblad et al., 2014; Trinity et al., 2011).

Various cycling body positions are reported within the literature including supine (body inline and parallel to the horizon), semi‐recumbent/recumbent (upper body inclined with the lower body parallel to the horizon) and semi‐upright (body inline at ∼40° to the horizon). In comparison to exercise in an upright position, supine submaximal exercise results in an elevated increase in cardiac output (CO) arising from enhanced stroke volume (SV) despite a blunted increase in heart rate (HR) (Hughson et al., 1991; Leyk et al., 1994; Sundblad et al., 2014). Submaximal rates of oxygen consumption (${\dot{V}}_{O_{2}}$) have been reported to be unaffected (Leyk et al., 1994; MacDonald et al., 1998; Terkelsen et al., 1999) or reduced (Armour et al., 1998; Terkelsen et al., 1999) in the supine compared to upright position. In addition, impaired lower limb perfusion when supine yields reduced active skeletal muscle oxygen saturation ($S_{mO_{2}}$) (Denis & Perrey, 2006; DiMenna, Bailey, & Jones, 2010, DiMenna, Wilkerson, Burnley, et al., 2010; Jones et al., 2006), perhaps contributing to premature fatigue. Of note, impaired lower limb perfusion during moderate intensity cycling is not ameliorated by a prior bout of priming exercise suggesting that priming‐induced elevations in blood flow are matched to increased oxygen utilization (DiMenna, Wilkerson, Burnley, et al., 2010), though the abolishment of a slow component is present (Jones et al., 2006). Despite the application of semi‐upright exercise during assessments of stress echocardiography (Sicari et al., 2008) and exercise right heart catheterization (Bentley et al., 2020), the physiological effects are poorly described, as a supine exercise position has been the primary area of previous investigations. Evidence suggests no difference in submaximal ${\dot{V}}_{O_{2}}$ (Quinn et al., 1995; Scott et al., 2006; Wehrle et al., 2021) between semi‐recumbent and upright submaximal exercise, yet maximal ${\dot{V}}_{O_{2}}$ is reduced (Dillon et al., 2021). Anecdotal evidence in our experience suggests that submaximal exercise in a semi‐upright position is perceived as more challenging, perhaps aligning with the perception from higher intensity semi‐recumbent exercise (Walsh‐Riddle & Blumenthal, 1989), although this has yet to be confirmed. To date, no study has explored the effect of a semi‐upright body position on the integrative cardiovascular, skeletal muscle and ventilatory responses to submaximal exercise. Establishing this foundational knowledge is necessary for appropriate interpretation of physiological measures during semi‐upright exercise.

Therefore, the purpose of this study was to determine the effect of a semi‐upright body position on the cardiovascular, skeletal muscle and ventilatory responses to submaximal exercise. We hypothesized that a semi‐upright cycling position, a position akin to that of common cardiac and cardiopulmonary assessments, would not impact submaximal ${\dot{V}}_{O_{2}}$, although CO would be elevated compared to an upright position. Despite the maintenance of ${\dot{V}}_{O_{2}}$ and an elevated CO, we further hypothesized reduced $S_{mO_{2}}$ and greater perceived exertion compared to an upright exercise.

## 2 METHODS

### 2.1 Ethical approval

The University of Toronto Health Sciences research ethics board approved this study (#41432) according to the terms of the latest version of the *Declaration of Helsinki*, and all participants provided written informed consent before their participation.

### 2.2 Participants

Twenty healthy, recreationally active (<3 h/week of structured exercise) individuals participated in this study (22 ± 3 years, 50% female) between 28 February and 26 July 2022. Health status was confirmed by the completion of the Physical Activity Readiness Questionnaire for Everyone (PAR‐Q+) (Jamnik et al., 2011). Participants were excluded if they were unable to perform cycling exercise, regularly used tobacco, had a vastus lateralis adipose tissue thickness greater than 12.5 mm, had any prior history of cardiovascular disease or did not adhere to University of Toronto's COVID‐19 protocols. During study participation, no participants were infected with COVID‐19 and none had required a COVID‐19 related hospitalization previously.

### 2.3 Study design

This was a within‐participant study in which submaximal cycling exercise was completed in two different body positions in order to manipulate haemodynamic conditions during exercise. Neither participants nor experimenters were blinded to the two exercise body positions. Participants reported to the laboratory for a single data collection session. Participants were asked to refrain from exercise for 24 h, caffeine and/or any energy‐altering substances for 12 h, and food for 4 h prior to their laboratory visit.

### 2.4 Submaximal cycling exercise

**FIGURE 1:**
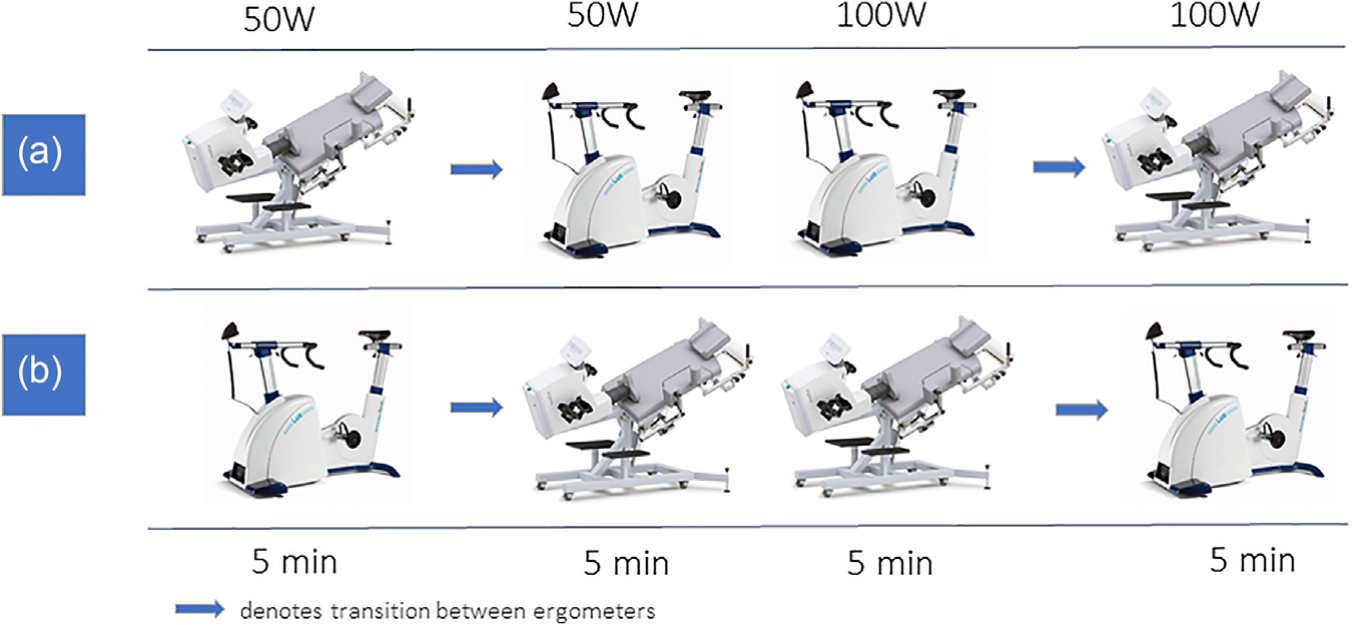
Experimental protocol. (a) Exercise initiated in the semi‐upright (40° incline) position. (b) Exercise initiated in the upright position. Participants completed four alternating bouts of submaximal cycling at 50 and 100 W each for 5 min. Exercises shown in (a) and (b) were randomized and counterbalanced. Prior to exercise, 2 min of rest was completed in both a seated position and on the ergometer in the initial exercise position.

Participants completed four alternating bouts of submaximal cycling. Cycling at each of 50 and 100 W was completed for 5 min on both a standard upright ergometer (Excalibur Sport, Lode BV, Groningen, Netherlands) and a semi‐upright tilt‐recline table ergometer (40° incline with no lateral tilt; Ergoline, Ergoselect 1200E, Bitz, Germany). In total, 20 min of submaximal exercise was completed and the initial ergometer position was randomized and counterbalanced (Figure 1). Seat position was adjusted beforehand for each ergometer to ensure similar knee angles and participant comfort. Pedalling cadence was self‐selected at a pace between 60 and 80 rpm and held constant across intensities and body position within a participant. Transitions between ergometers were completed within 1 min. Prior to exercise, 2 min of rest was completed in both a seated upright position in a chair and on the ergometer in the initial exercise position (i.e., upright or semi‐upright body position).

To assess ${\dot{V}}_{O_{2}}$ at the same power (in watts) between the two ergometers during submaximal cycling, a supine cycling position was employed. On a separate day, a subset of participants (*n* = 4, 25% female) completed 5 min of consecutive supine cycling at 50 and 100 W using both the standard upright ergometer and the semi‐upright tilt‐recline table ergometer. Each session was separated by at least 1 h. Participants were positioned with their backs on the floor behind the upright ergometer, with the ergometer elevated such that the distance from the pedal at the 12 o'clock position to the floor was 53.3 cm. This height matched the distance from the pedal at the 12 o'clock position to the table of the semi‐upright tilt‐recline table ergometer when in the supine position. Participants were outfitted as noted above.

### 2.5 Standard anthropometric data

Upon arrival at the laboratory, standard anthropometric measurements were obtained from each participant. Age, height and weight were obtained as well as adipose tissue thickness of the right vastus lateralis ∼14 cm above the patella. A 7‐day physical activity questionnaire adapted from Sarkin et al. (1997) was completed to confirm current physical activity habits.

### 2.6 Central haemodynamics

A finger photoplethysmograph was placed on the middle finger of the right hand with the arm secured by a Rolyan buckle closure arm sling and placed on the chest over the heart and kept in this position throughout the study. This device provides beat‐by‐beat estimates of SV and thereby computed CO and total peripheral resistance (TPR) via the ModelFlow (Finometer MIDI, Finapres Medical Systems, Enschede, the Netherlands). This device provides relative changes in these variables. In addition, this device provides measures of mean arterial blood pressure (MAP). All variables were measured continuously.

### 2.7 Skeletal muscle saturation

Skeletal muscle oxygen saturation of the right vastus lateralis was measured by continuous wave near‐infrared spectroscopy (Moxy NIRS; Fortiori Design LLC, Hutchinson, MN, USA) at 2 Hz. This device has a coefficient of variation during 100 W of cycling of 4% (Crum et al., 2017) and a penetration depth of 12.5 mm based upon light detector and emitter distances. The device was secured to the skin over the vastus lateralis ∼14 cm above the patella with adhesive tape and an elastic bandage (Bentley et al., 2018). This device provides relative changes in the proportional concentrations of oxygenated and deoxygenated haemoglobin/myoglobin ($S_{mO_{2}}$ = oxy‐[haemoglobin/myoglobin]/total [haemoglobin/myoglobin], %). $S_{mO_{2}}$ was measured continuously.

### 2.8 Pulmonary gas exchange and oxygen consumption

Pulmonary gas exchange and ventilation were measured breath‐by‐breath using a calibrated, computer‐based system (Vmax Encore 229, CareFusion, Yorba Linda, CA, USA). Pulmonary gas exchange was measured continuously. Due to COVID‐19, a MicroGard type B bacterial/viral filter was placed inline such that all inspired and expired air passed through the filter.

### 2.9 HR and ratings of perceived exertion

Participants were outfitted with a three‐lead electrocardiogram for the measurement of HR throughout exercise. At rest and 4 min into each exercise intensity, ratings of whole‐body perceived exertion (RPE) and breathlessness (dyspnoea) were obtained in succession on the Borg 6–20 scale. Prior to exercise participants were oriented to the scale and read a script modified from Faulkner & Eston (2007).

### 2.10 Data analysis

#### 2.10.1 Oxygen consumption

${\dot{V}}_{O_{2}}$ was obtained on a breath‐by‐breath basis. At rest and each exercise intensity, a 30 s average was computed within the final minute. During supine cycling (*n* = 4), ${\dot{V}}_{O_{2}}$ was obtained on a breath‐by‐breath basis with a 30 s average computed within the final minute of each exercise intensity.

#### 2.10.2 Stroke volume, CO, mean arterial pressure and skeletal muscle saturation

SV, CO, MAP and $S_{mO_{2}}$ were averaged over 30 s within the final minute of rest and each exercise intensity.

### 2.11 Statistical analysis

All statistics were calculated using a combination of SPSS 28 (IBM Corp., Armonk, NY, USA) and SigmaPlot 12.0 (Systat Software, Inc., San Jose, CA, USA). Normality of data was assessed visually with Q‐Q plots and quantitatively with a Shapiro–Wilk test. To explore the effect of body position, sex and exercise intensity on all reported variables, a three‐way repeated measures analysis of variance (ANOVA) was completed. Assumptions of normality and homogeneity of variance in the repeated measures ANOVA were met. Following significant *F*‐statistics, Bonferroni corrected *post hoc* tests were completed. A significant three‐way interaction was not present, so subsequent two‐way repeated measures ANOVA (body position by sex; body position by intensity; sex by position) followed by Bonferroni corrected *post hoc* tests were not needed. Bland–Altman analysis was completed for supine exercise (*n* = 4) with the submaximal ${\dot{V}}_{O_{2}}$ at each intensity compared using a Bonferroni‐corrected paired Student's *t*‐test. Parametric data are presented as means ± standard deviation. Non‐parametric data are presented as median (interquartile range, Q1–Q3). Statistical significance was set at *P *< 0.05 for all analyses. Absolute data are reported at rest in the seated resting baseline position, while Δ from seated baseline is reported during exercise.

## 3 RESULTS

### 3.1 Participant characteristics and seated baseline

Standard anthropometric information is presented in Table 1. Males were taller (*P* = 0.001), heavier (*P* = 0.03) and had lower adipose tissue thickness at the vastus lateralis (*P *< 0.001) compared to females. Seated baseline data are presented in Table 2.

**TABLE 1:** Anthropometric measures.

Variable	All (*n* = 20)	Male (*n* = 10)	Female (*n* = 10)
Age (years)	22 ± 3	23 ± 4	21 ± 1
Height (m)	1.70 ± 0.08	1.75 ± 0.07	1.65 ± 0.05*
Weight (kg)	73 ± 13	79 ± 14	66 ± 10*
BMI (kg/m^2^)	25.0 ± 4.0	25.6 ± 4.1	24.4 ± 4.0
Adipose tissue thickness (mm)	7.0 ± 2.6	5.2 ± 1.8	8.8 ± 1.8*
7‐day PAR score (METs/week)	271 ± 27	276 ± 26	268 ± 27

*Note*: Parametric variables are presented as means ± SD. ^*^Statistically significant difference between males and females (*P *< 0.05). Abbreviations: BMI, body mass index; PAR, physical activity recall.

**TABLE 2:** Seated baseline measures.

Variable	All (*n* = 20)	Male (*n* = 10)	Female (*n* = 10)
${\dot{V}}_{O_{2}}$ (L/min)	0.28 ± 0.08	0.30 ± 0.08	0.26 ± 0.07
${\dot{V}}_{E}$ (L/min)	10 ± 2	10 ± 2	10 ± 2
*V*_T_ (L)	0.66 ± 0.17	0.69 ± 0.14	0.64 ± 0.20
*f*_B_ (breaths/min)	16 ± 4	15 ± 4	16 ± 5
HR (bpm)	78 ± 14	81 ± 13	76 ± 15
MAP (mmHg)	99 ± 15	104 ± 15	95 ± 14
RPE	6 (6–6)	6 (6–6)	6 (6–6)
Dyspnoea	6 (6–6)	6 (6–6)	6 (6–6)

*Note*: Parametric variables are presented as means ± SD. Non‐parametric variables are presented as median (interquartile range, Q1–Q3). Abbreviations: *f*_B_, breathing frequency; HR, heart rate; MAP, mean arterial blood pressure; RPE, rating of perceived exertion; ${\dot{V}}_{E}$, minute ventilation; ${\dot{V}}_{O_{2}}$, rate of oxygen consumption; *V*_T_, tidal volume.

### 3.2 Pulmonary gas exchange

**FIGURE 2:**
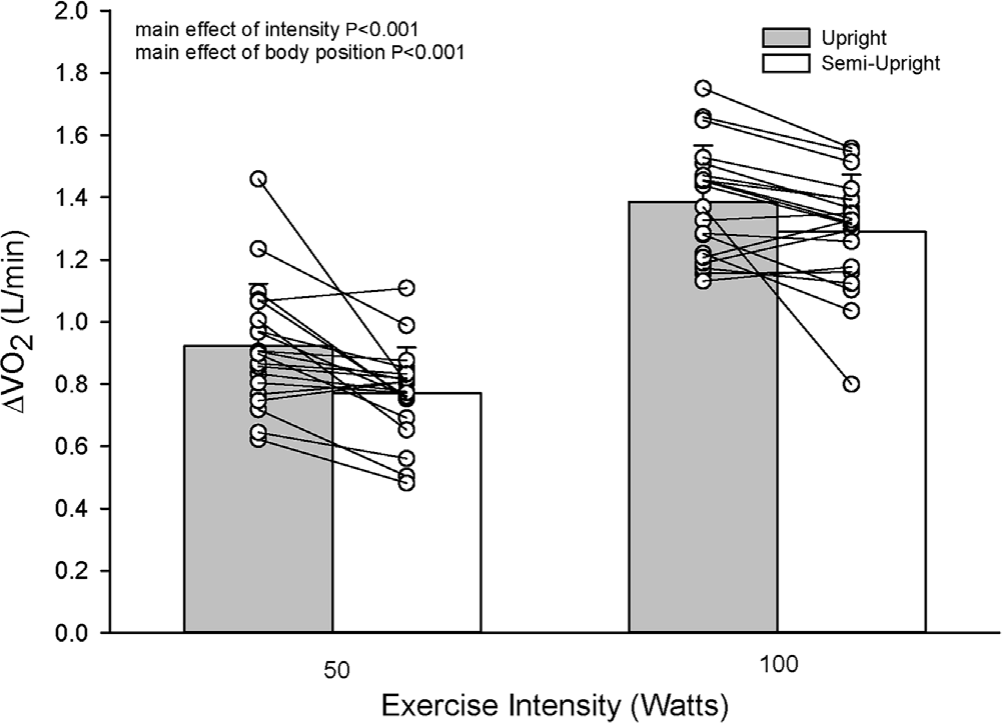
Rate of oxygen consumption. Rate of oxygen consumption during upright (grey bare) and semi‐upright (white bare) submaximal cycling exercise. Individual responses are shown by open circles and connected by lines between body positions (*n* = 20).

**FIGURE 3:**
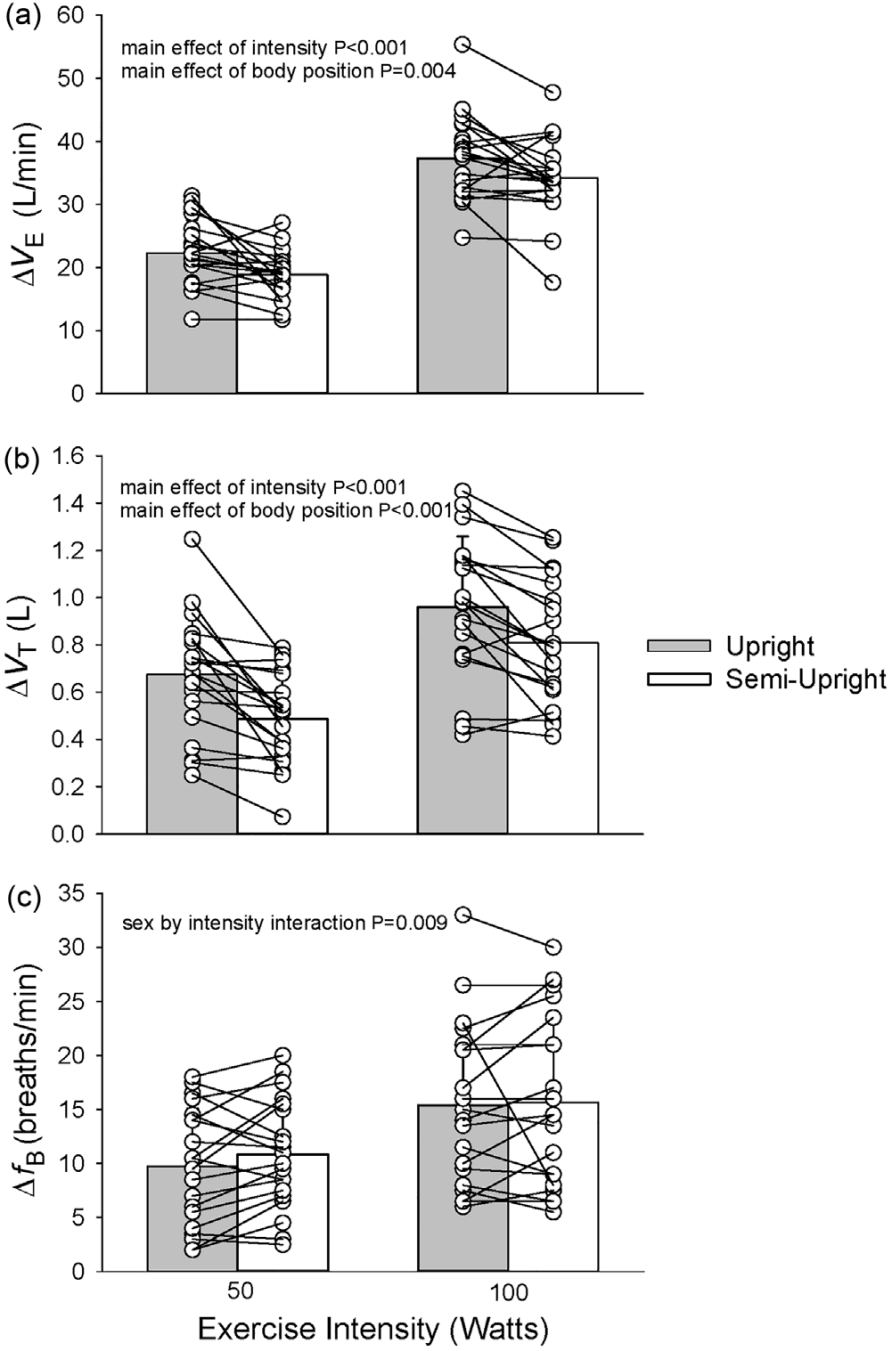
Pulmonary ventilation. (a) Minute ventilation. (b) Tidal volume. (c) Breathing frequency. Pulmonary ventilation and constituents during upright (grey bar) and semi‐upright (white bar) submaximal cycling exercise. Individual responses are shown by open circles and connected by lines between body positions (*n* = 20).

There was a main effect of intensity and body position on ${\dot{V}}_{O_{2}}$ with a greater increase at 100 W compared to 50 W (1.337 ± 0.156 vs. 0.847 ± 0.143 L/min, *P *< 0.001, η_p_^2^ = 0.956) and an elevated ${\dot{V}}_{O_{2}}$ in the upright compared to semi‐upright position (1.154 ± 0.165 vs. 1.030 ± 0.130 L/min, *P *< 0.001, η_p_^2^ = 0.565) (Figure 2). A main effect of intensity was also present for minute ventilation (${\dot{V}}_{E}$) with a greater increase at 100 W compared to 50 W (36 ± 6 vs. 21 ± 4, *P *< 0.001 L/min, η_p_^2^ = 0.934), while a main effect of body position revealed a greater increase in the upright compared to semi‐upright position (30 ± 6 vs. 27 ± 5 L/min, *P* = 0.004, η_p_^2^ = 0.369). A main effect of intensity on tidal volume (*V*_T_) identified a greater increase at 100 W compared to 50 W (0.88 ± 0.21 vs. 0.58 ± 0.17 L, *P *< 0.001, η_p_^2^ = 0.897) and a greater increase in the upright compared to semi‐upright position (0.82 ± 0.21 vs. 0.65 ± 0.18 L, *P *< 0.001, η_p_^2^ = 0.634). There was an interaction of sex and intensity (*P* = 0.009, η_p_^2^ = 0.320) on breathing frequency (*f*_B_) with females having a greater *f*_B_ at 50 W (13 ± 4 vs. 8 ± 4 breaths/min, *P* = 0.015) and 100 W (20 ± 6 vs. 11 ± 6 breaths/min, *P* = 0.004). Both males and females had greater *f*_B_ at 100 W compared to 50 W (*P *< 0.001) (Figure 3).

### 3.3 Central haemodynamics and skeletal muscle saturation

**FIGURE 4:**
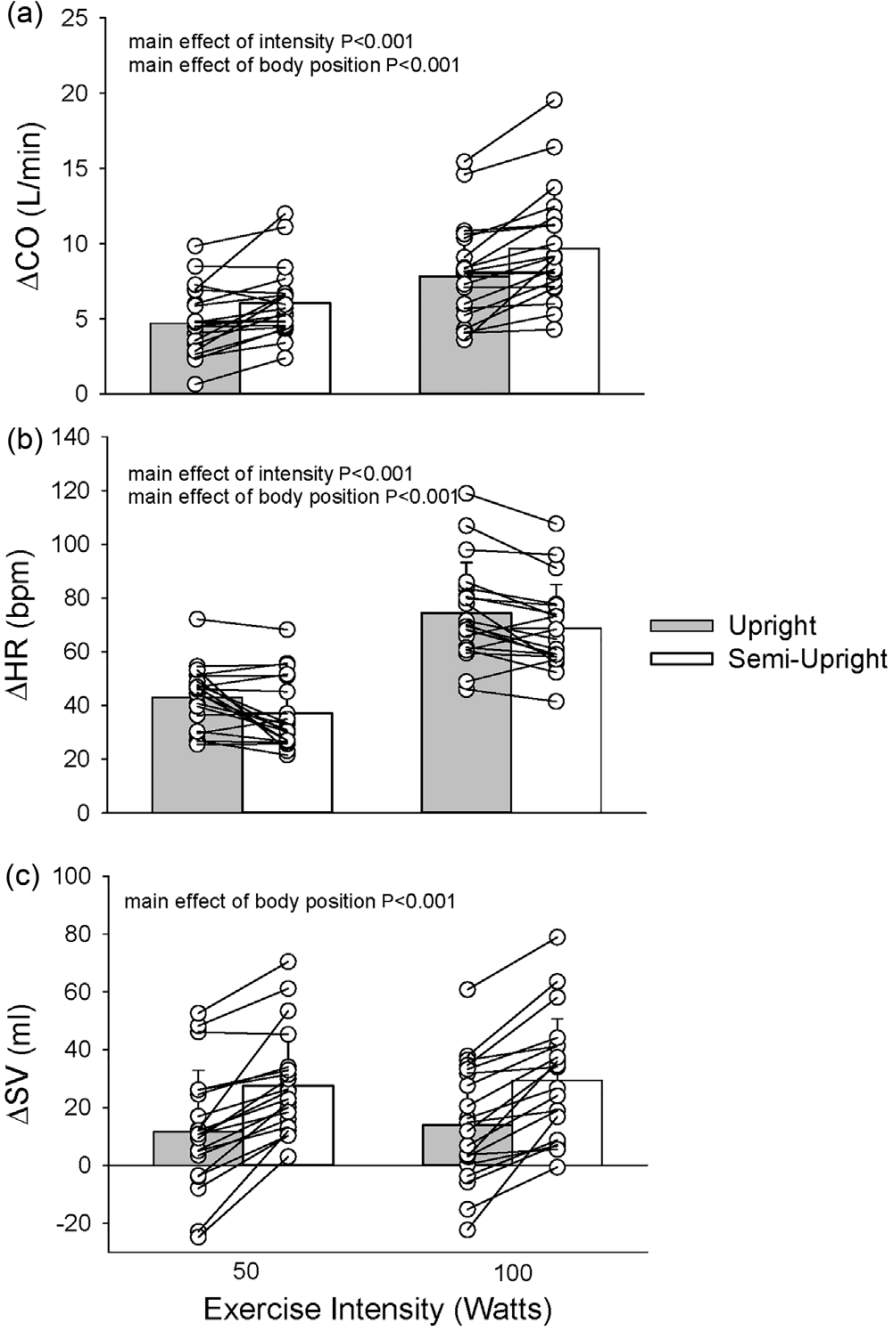
Central haemodynamics. (a) Cardiac output. (b) Heart rate. (c) Stroke volume. Central haemodynamics during upright (grey bar) and semi‐upright (white bar) submaximal cycling exercise. Individual responses are shown by open circles and connected by lines between body positions (*n* = 20).

There was a main effect of intensity on CO with a greater increase at 100 W compared to 50 W (8.9 ± 3.3 vs. 5.5 ± 2.1 L/min, *P *< 0.001, η_p_^2^ = 0.823). There was also a main effect of body position with a greater increase in CO during semi‐upright compared to upright cycling (7.9 ± 2.8 vs. 6.4 ± 2.6 L/min, *P *< 0.001, η_p_^2^ = 0.663). This increase in CO was driven by an increase in HR (50 W vs. 100 W: 40 ± 12 vs. 71 ± 16 bpm, *P *< 0.001, η_p_^2^ = 0.900) as SV was unaffected by exercise intensity (*P* = 0.247, η_p_^2^ = 0.074). Both HR and SV had a main effect of body position, with the increase in HR being greater in the upright position (59 ± 13 vs. 52 ± 13 bpm, *P *< 0.001, η_p_^2^ = 0.572) and the increase in SV being reduced (14 ± 20 vs. 29 ± 19 mL, *P *< 0.001, η_p_^2^ = 0.822) compared to semi‐upright (Figure 4).

There was an intensity and body position interaction on MAP (*P* = 0.045, η_p_^2^ = 0.205). Within the upright position, there was no difference between the increase in MAP at 50 and 100 W (18 ± 15 vs. 20 ± 12 mmHg, *P* = 0.482), but within the semi‐upright position, the increase in MAP was less at 50 W compared to 100 W (6 ± 7 vs. 14 ± 14 mmHg, *P *< 0.001). At both 50 W (18 ± 15 vs. 6 ± 7 mmHg, *P *< 0.001) and 100 W (20 ± 14 vs. 14 ± 12 mmHg, *P *< 0.001) the increase in MAP was greater in the upright compared to semi‐upright body position (data not shown).

There was a main effect of both intensity and body position on TPR. The reduction in TPR was less at 50 W compared to 100 W (−7.7 ± 4.8 vs. −9.8 ± 5.5 mmHg/L/min, *P *< 0.001, η_p_^2^ = 0.603) and the reduction was blunted in the upright compared to semi‐upright position (−7.8 ± 5.2 vs. −9.7 ± 5.1 mmHg/L/min, *P *< 0.001, η_p_^2^ = 0.680) (data not shown).

**FIGURE 5:**
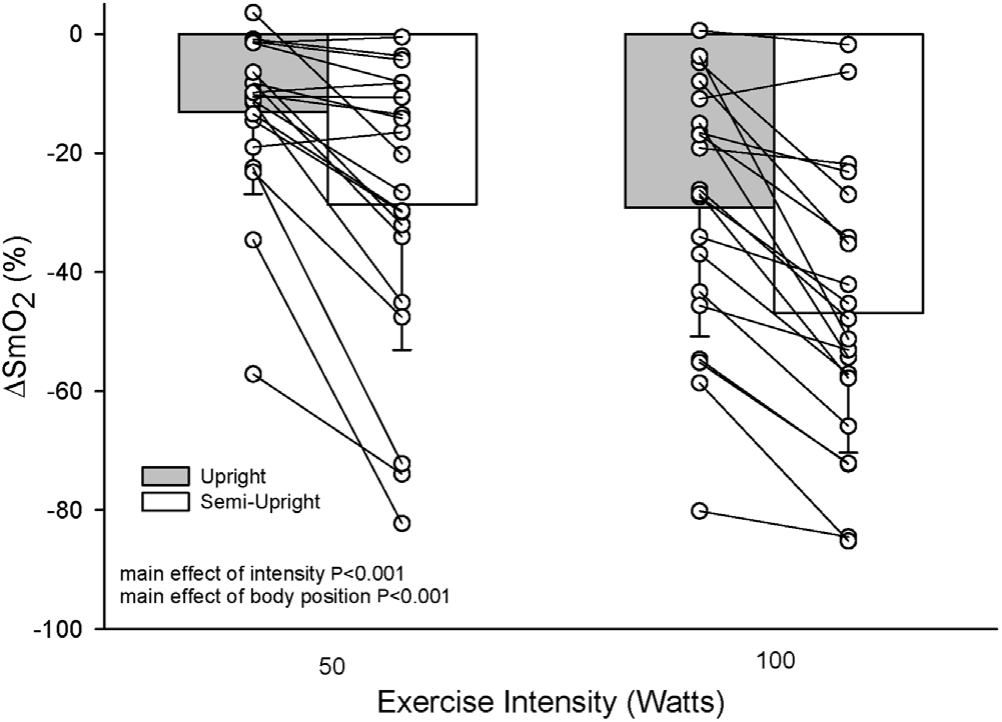
Skeletal muscle saturation. Skeletal muscle saturation during upright (grey bar) and semi‐upright (white bar) submaximal cycling exercise. Individual responses are shown in open circles and connected by solid lines between body positions (*n* = 20).

There was a main effect of both intensity and body position on $S_{mO_{2}}$. The reduction in $S_{mO_{2}}$ was less at 50 W compared to 100 W (−21 ± 19 vs. −38% ± 22%, *P *< 0.001, η_p_^2^ = 0.814) and less in the upright compared to semi‐upright body position (−21 ± 18 vs. −38% ± 23%, *P *< 0.001, η_p_^2^ = 0.711) (Figure 5).

### 3.4 Perceived exertion and dyspnoea

There was an intensity by sex interaction on RPE (*P* = 0.018, η_p_^2^ = 0.271). While RPE at both 50 W (male vs. female; 9 (8–10) vs. 8 (7–10), *P* = 0.749) and 100 W (13 (11–14) vs. 13 (12–14), *P* = 0.296) did not differ, RPE was greater at 100 W compared to 50 W within males (*P *< 0.001) and females (*P *< 0.001). There was a main effect of body position on RPE with the upright body position having a lower RPE compared to the semi‐upright position (10 (8–12) vs. 11 (9–13), *P* = 0.013, η_p_^2^ = 0.295). Dyspnoea also had an intensity by sex interaction (*P* = 0.011, η_p_^2^ = 0.308) with dyspnoea at 50 W not different between males and females (8 (7–9) vs. 9 (7–9), *P* = 0.648), but at 100 W, dyspnoea was reduced in males compared to females (11 (10–12) vs. 13 (12–13), *P* = 0.043). Dyspnoea also had a main effect of body position with dyspnoea being lower in the upright compared to semi‐upright position (10 (8–12) vs. 11 (10–13), *P* = 0.020, η_p_^2^ = 0.265) (data not shown).

### 3.5 Oxygen consumption between ergometers in the supine position

There was no difference in ${\dot{V}}_{O_{2}}$ between the standard upright ergometer and the semi‐upright tilt‐recline table ergometer in the supine exercise position at either 50 W (0.010 ± 0.004 L/min, *P* = 0.986) or 100 W (0.069 ± 0.004 L/min, *P* = 0.518). Bland–Altman analysis revealed an overall bias of −0.0108 L/min with the semi‐upright tilt‐recline table ergometer, and a 95% bias confidence interval of −0.046 to 0.024 L/min.

## 4 DISCUSSION

The application of exercise in a semi‐upright body position is common when exploring cardiac function. While previous work explored individual parameters of cardiac, pulmonary or skeletal muscle responses, no such work has provided an integrative assessment resulting in an incomplete knowledge of the underlying physiology. This foundational knowledge is necessary for appropriate interpretation of physiological measures during semi‐upright exercise. The primary findings from the present investigation revealed: (1) exercise in the semi‐upright body position results in a blunted increase in ${\dot{V}}_{O_{2}}$ at the same exercise intensity, (2) the increase in CO is greater during exercise in a semi‐upright position, yet $S_{mO_{2}}$ was r, and (3) the increase in perceived exertion and dyspnoea during exercise are greater in a semi‐upright position. The results from this study provide important information about the application and interpretation of physiological responses in a semi‐upright exercise position that should be considered when extrapolating information to more common exercise positions.

### 4.1 Pulmonary oxygen consumption and ventilation

In the present study, the completion of semi‐upright exercise was facilitated by the application of a purpose‐built, tilt‐recline table ergometer. This ergometer is specifically designed for application during stress echocardiography and exercise right heart catheterization. As a result, hip and knee joint angles were similar between the two cycling positions as the body remains inline. We observed a blunted increase in submaximal ${\dot{V}}_{O_{2}}$ during semi‐upright exercise. Previous work utilizing a similar specialized ergometer (∼40° incline) observed reductions in peak ${\dot{V}}_{O_{2}}$ when semi‐upright (Dillon et al., 2021; Wehrle et al., 2021), yet no difference at 70 W during a step‐incremental exercise protocol (Wehrle et al., 2021) compared to cycling upright. Other studies using custom‐built ergometers at 120° from the horizon (Scott et al., 2006) or 65° and 30° recumbent postures (Egana et al., 2010) found no difference in submaximal ${\dot{V}}_{O_{2}}$ compared to upright cycling. While previous studies (Dillon et al., 2021; Wehrle et al., 2021) utilized similar cycling equipment, they differed in the experimental methodology, and as such, preclude a clear comparison to the present study. The reduced submaximal steady‐state ${\dot{V}}_{O_{2}}$ in the present study may arise due to a reduced ${\dot{V}}_{O_{2}}$ stemming from changes in active skeletal muscle efficiency or reduced overall whole‐body muscle activation. That being said, we feel the latter may be more likely. Within both cycling positions, the participant's right arm was held by an arm sling on the chest over their heart to ensure finger photoplethysmography measures were obtained in a similar relative position to heart level. When semi‐upright, this did not impact cycling as the participant's back was fully supported given the inclined position. When upright, however, the participant's hands were not on the handlebars providing support and as a result, activation of the erector spinae to maintain an upright posture (Shima et al., 2022) may have slightly elevated ${\dot{V}}_{O_{2}}$ in the upright position compared to semi‐upright. Subsequently, the purpose‐built tilt‐recline table ergometer may have unloaded said postural muscles by fully supporting the back resulting in a lower ${\dot{V}}_{O_{2}}$ such that when compared, the blunted increase in ${\dot{V}}_{O_{2}}$ in the semi‐upright position was observed.

The present study observed an attenuated increase in ${\dot{V}}_{E}$, which arose from an attenuated increase in *V*_T_ as *f*_B_ was unaffected by body position. Wehrle et al. (2021) also observed reductions in ${\dot{V}}_{E}$ and *V*_T_ at submaximal exercise thresholds and this aligns with blunted ventilatory responses during supine exercise in comparison to upright (Armour et al., 1998; Hughson et al., 1991; Terkelsen et al., 1999). Interestingly, Walsh‐Riddle and Blumenthal (1989) observed increases in submaximal ${\dot{V}}_{E}$ in a semi‐upright position compared to upright, but only in females. We did not observe a sex effect in the present study and this previous observation may arise due to the older (34–62 years of age) cohort and potential disparate ageing effects between sexes on type I muscle fibres (Roberts et al., 2018).

### 4.2 Central haemodynamics and skeletal muscle saturation

As expected, moving from an upright to semi‐upright exercise position increased submaximal CO through an elevation in SV. Transitioning from exercise in the supine to 70° head‐up tilt position produces reductions in SV due to impaired cardiac filling and reductions in end‐diastolic volume (Cotsamire et al., 1987). Indeed, when transitioning from upright to supine, elevations in CO due to increases in SV are observed (Hughson et al., 1991). While elevations in CO have been observed with supine exercise, both elevations in leg blood flow during supine exercise compared to sitting (Cronestrand, 1970) and reductions in the transient blood flow response without compromises in steady state blood flow (MacDonald et al., 1998) have been observed. The implications of body position on active muscle blood flow are an important consideration given the unique haemodynamic position imposed by gravity (Hinghofer‐Szalkay, 2011). This is supported by the reduced reliance on the skeletal muscle pump to facilitate venous return in a supine position compared to upright (Takahashi et al., 2005). As a result, central cardiac filling may be enhanced due to improved venous return and elevated preload, though the local perfusion pressure at the active skeletal muscle (i.e., vastus lateralis) may be impaired.

We observed greater active muscle desaturation in the presence of an elevated CO. In the absence of measures of skeletal muscle blood flow, it is difficult to identify where exactly this elevation in central flow is perfusing. With rhythmic muscular contraction, as is the case with cycling, the venous compartment is continuously emptied and venous pressure approximates zero (Folkow et al., 1970). This elevates the local pressure gradient for blood flow during relaxation in between contractions. The ability to empty venous volume would occur independent of body position and thus semi‐upright exercise challenges local muscle blood flow due to a reduction in local arterial pressure arising from modulation of a hydrostatic column between the heart and the vastus lateralis. We calculated the pressure difference between the heart (3rd costal space) and the vastus lateralis (at the measurement site of $S_{mO_{2}}$). When the knee is flexed, with feet in the pedals, local perfusion pressure at the vastus lateralis is reduced in the semi‐upright compared to the upright position by ∼30 mmHg (+3 ± 2 vs. +33 ± 8 mmHg, *P *< 0.001, *n* = 5). Indeed, the application of lower body negative pressure during supine resistance exercise improves skeletal muscle oxygenation due to an improved local pressure gradient for blood flow (Parganlija et al., 2019). In the absence of experimental improvements in local oxygen delivery, supine exercise results in greater deoxygenation (DiMenna, Bailey & Jones, 2010; Goulding, Marwood, Okushima, et al., 2020, 2020, Goulding, Okushima, Marwood, et al., 2020; Goulding et al., 2021). Interestingly, this greater deoxygenation appears limited to superficial muscle groups as perfusion to deep muscle appears unaffected (Goulding, Marwood, Okushima et al., 2020). As blood flow is the product of a pressure gradient across a vascular bed and the vascular conductance (reciprocal of resistance) of said vasculature, within the context of the impaired oxygenation and subsequent greater desaturation at the vastus lateralis in the semi‐upright exercise position, this position also yielded a greater increase in MAP and a greater blunting of TPR perhaps to mitigate the initial challenge in local perfusion pressure induced by postural manipulation.

### 4.3 Perceived exertion and dyspnoea

An important consideration when incorporating semi‐upright exercise into assessments of cardiopulmonary function is the perceived effort during exercise as this subjective rating is an important determinant of exercise tolerance and our ability to perform activities of daily living. Expectedly, the perception of effort at maximal exercise is not different between body positions (Wehrle et al., 2021) as it can be appreciated that the cessation of exercise, whether at differing exercise intensities or ${\dot{V}}_{O_{2}}$, will occur at similar subjective levels of exertion. In the present study, we observed elevated ratings of perceived exertion as well as dyspnoea when in the semi‐upright cycling position. This observation contrasts previous work in older (∼50–70 years of age) individuals when comparing upright to supine submaximal exercise in which either no difference in dyspnoea (Armour et al., 1998) or RPE (Quinn et al., 1995) was observed, or a greater RPE was observed in the upright compared to the supine position (Walsh‐Riddle & Blumenthal, 1989). That being said, leg specific RPE has been reported to be greater in the supine compared to recumbent position (Kato et al., 2011). Inferring a greater leg RPE in semi‐upright compared to upright cycling, it may be that our participants placed a greater leg effort on their overall RPE compared to previous studies. Further, given the reduced $S_{mO_{2}}$ at the same exercise intensity in the semi‐upright position, it may be that greater disruptions in intracellular skeletal muscle homeostasis in light of apparent impaired convective oxygen delivery may manifest in the elevated RPE and dyspnoea presently observed. Nonetheless, our anecdotal observation of exertion from previous participants aligns with the results from the present study.

### 4.4 Practical implications and experimental considerations

Results from the present study are directly transferable to the application of stress echocardiography (Sicari et al., 2008) and exercise right heart catheterization (Bentley et al., 2020) in which assessments of cardiac function and the contribution of pulmonary vasculature to disease occur during aerobic exercise. It is not uncommon for upright interventions, including cardiopulmonary exercise testing, to be applied in patients with cardiovascular disease (Albouaini et al., 2007). Our results suggest that exercise in a semi‐upright position seems to compromise peripheral muscle perfusion despite enhanced central output. While it may be hypothesized from previous work that rest to exercise elevations in CO primarily perfuse active muscle (Armstrong et al., 1987), the disparate haemodynamic effects on lower limb perfusion cannot be overlooked. Further, the observed difference in the subjective perception of effort and dyspnoea in our study, both important contributors to the completion of activities of daily living and by extension quality of life, highlight the need to consider postural effects. As such, an integrative approach considering the implications of exercising body position should be considered when completing detailed assessments of cardiac function and the contribution of pulmonary vasculature to disease.

We acknowledge that the use of absolute exercise intensities may result in differences between participants in muscle recruitment and activation, alongside oxygen demand per unit of active muscle mass. These differences may influence cardiovascular, skeletal muscle and ventilatory responses to submaximal cycling between participants; however, a strength of the present design was the randomized and counterbalanced experimental protocol in which each participant effectively served as their own control. While doing so, in order to obtain consistency in the assessments of central haemodynamics, the arm was held in place on the chest over the heart. This may have prevented a typical upright cycling experience in which the arms provide support via placement on the handlebars. While care was taken to ensure similar exercise conditions between body positions (e.g., seat height and knee angles, participant comfort), we were unable to confirm similar skeletal muscle activation of postural muscles as well as active leg muscles between body positions in the absence of electromyographic measures of muscle recruitment. It may be that differences in skeletal muscle motor recruitment between positions is an important consideration. Further, in the absence of unloaded cycling, we are unable to confirm if there is a lower oxygen cost between body positions associated with simply moving the legs. As such, a potential difference in unloaded cycling oxygen cost may have contributed to the attenuated ${\dot{V}}_{O_{2}}$ when in the semi‐upright position. Despite the use of calibrated ergometers, it is possible that the actual power (in watts) may have differed; however, subset analysis (*n* = 4) in the supine cycling position revealed no difference between ergometers with a bias of just −0.0108 L/min. As such, it is not believed that a potential power difference influenced the interpretation of results. Lastly, while local muscle oxygenation was assessed, muscle blood flow was not measured due to technical limitations in the application of ultrasound during cycling at the femoral artery given hip movement. As such, it is difficult to identify the local blood flow response between cycling positions in the presence of disparate CO, $S_{mO_{2}}$ and local perfusion pressures.

### 4.5 Conclusion

The completion of exercise in a semi‐upright body position is common when assessing cardiac function and the contribution of pulmonary vasculature to disease. While semi‐upright submaximal cycling produces a greater elevation in CO, this elevation does not seem to perfuse the active skeletal muscle as $S_{mO_{2}}$ was reduced more more compared to upright cycling. Further, despite an attenuated increase in ${\dot{V}}_{O_{2}}$ in the semi‐upright position, perceived exertion and dyspnoea are elevated. These considerations are important when interpreting physiological responses in a semi‐upright exercise position and future work should explore skeletal muscle activation and lower limb blood flow in these two exercise positions.

## AUTHOR CONTRIBUTIONS

Robert F. Bentley contributed to the conception or design of the work, acquisition, analysis, or interpretation of data for the work and drafting of the work or revising it critically for important intellectual content. Jonaline B. Bernal, Daniel C. Basile, Adam N. Di Salvo, and Jacob L. Schwartz contributed to the acquisition, analysis, or interpretation of data for the work and drafting of the work or revising it critically for important intellectual content. All authors approved the final version of the manuscript and agree to be accountable for all aspects of the work in ensuring that questions related to the accuracy or integrity of any part of the work are appropriately investigated and resolved. All persons designated as authors qualify for authorship, and all those who qualify for authorship are listed.

## CONFLICT OF INTEREST

The authors declare no conflicts of interest. The results of the study are presented clearly, honestly, and without fabrication, falsification, or inappropriate data manipulation.

## Supporting information



Raw data.

## Data Availability

The data that supports the findings of this study are available in the Supporting information of this article.
